# Robust and compact digital Lensless Holographic microscope for Label-Free blood smear imaging

**DOI:** 10.1016/j.ohx.2023.e00408

**Published:** 2023-02-25

**Authors:** Carlos Buitrago-Duque, Brayan Patiño-Jurado, Jorge Garcia-Sucerquia

**Affiliations:** School of Physics, Universidad Nacional de Colombia - Sede Medellin, A.A: 3840, Medellin 050034, Colombia

**Keywords:** Point-of-care testing, Digital Holographic Microscopy, Label-free Microscopy, Lensless Imaging

## Abstract

The lack of equipped healthcare infrastructure in isolated hard-to-reach zones exposes their population to a higher risk of complications in common diseases. With a timely diagnosis setting a life-altering difference, worldwide efforts have been conducted for the development of point-of-care testing (PoCT) with cost-effective devices. Among the most common interests in PoCT is the analysis of blood smear samples, as they can help to detect, diagnose, and monitor a wide range of diseases and disorders. With microscopy being the traditional tool for these analyses, a significative advance has been the development of cost-effective digital holographic microscopy systems, driven in part by its label-free imaging capabilities that waive the need for any sample preprocessing. Here, a robust and portable digital lensless holographic microscope, functionalized for the analysis of non-preprocessed blood smear samples in PoCT environments, is presented, and its viability is tested in the observation of red blood cells. The device uses an optical fiber with a cone-shaped tip instead of a pinhole, which ensures the sturdiness of the system and eliminates the need for challenging alignment. While the distances of the microscope can be tuned before fabrication, the herein-reported operational parameters are functionalized for the specific analysis of blood samples.

## Graphical abstract

**Figure:**
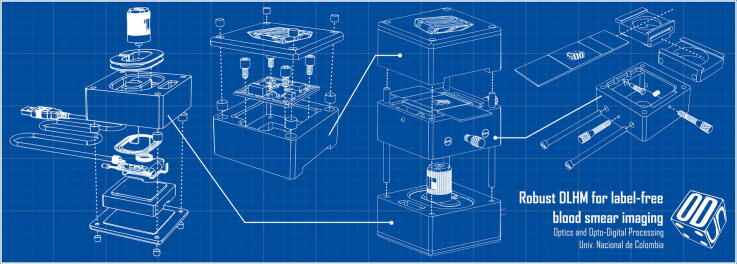

## Specifications table

**Table:** 

Hardware name	*Robust Digital Lensless Holographic Microscope for blood-smear imaging*
Subject area	Medical (e.g., pharmaceutical science)Biological sciences (e.g., microbiology and biochemistry)Educational tools and open-source alternatives to existing infrastructure
Hardware type	Imaging toolsField measurements and sensors
Closest commercial analog	Commercial Digital Holographic Microscopes, like the ones distributed by Lyncée Tec [1], Holmarc [2], Phi AB [3], Telight [4], or NanoLive [5].
Open-source license	Creative commons Attribution-ShareAlike 4.0 International (CC BY-SA 4.0)
Cost of hardware	250 USD
Source file repository	https://doi.org/10.17605/OSF.IO/4KET9

## Hardware in context

A timely diagnosis can set the difference between a life-threatening illness and a controlled affliction. Yet, access to equipped healthcare infrastructure in developing countries and low-resource settings is commonly limited to central cities and urban areas, leaving the rural regions and isolated hard-to-reach zones exposed to a higher risk of complications in common diseases. To fight this global health challenge, worldwide efforts have been conducted for the development and validation of point-of-care testing (PoCT) with cost-effective and portable devices for personalized medicine [6]. Among the most common interests in PoCT is the analysis of blood smear samples, as they can help to detect, diagnose, and monitor a wide range of diseases and disorders, including sickle-cell disease, anemia, and malaria [7]. For these analyses, optical microscopy inspection has been conventionally regarded as the gold standard in diagnosis [8]. However, optical microscopy has an elevated acquisition cost, is not portable, and requires the use of staining agents which further require specialized personnel and additional sample preparation time. In response, a significative advance in PoCT has been the development of cost-effective digital holographic microscopy systems [9], [10], [11], driven in part by its label-free imaging capabilities that waive the need for any sample processing before its observation, and the increase in mass production of consumer-grade digital electronics in the last decade that allows significant cost reductions in the acquisition of their main components.

Among such techniques, Digital Lensless Holographic Microscopy (DLHM) is one of the most simple architectures, constituting a modern realization of Gabor’s original invention [12]. As with any other holographic technique, it is a two-step imaging process. In the first stage, known as the recording, the sample under study is illuminated with a diverging spherical wavefront produced by a point source of light. The diffraction pattern that arises from this interaction is naturally magnified by free-space propagation and captured by a digital sensor [13]. The recorded image, which constitutes the hologram, can then be digitally processed in the second step, known as the reconstruction. In this stage, the complex-valued wavefield scattered by the sample can be reconstructed by computing the backwards diffraction process of the hologram multiplied by a converging spherical wavefront. From the resulting field, the sample’s intensity or phase distributions can then be promptly computed [14]. As can be read in the specialized literature regarding DLHM, from this information in-situ quantitative phase imaging can be achieved [15], permitting point-of-care diagnosis applications [16] and the retrieval of biological information [14], like dry-mass, biovolume, surface area, and cell dynamics, from the sample’s phase values. In this contribution, a robust, portable, and open-source DLHM functionalized for the analysis of non-preprocessed blood smear samples in PoCT environments, is presented and validated in the observation of red blood cells (RBCs).

Similar devices to the design herein proposed can be found in the literature. In particular, those reported by Tobón-Maya et al. [10] and Amann et al. [11] present notable results in the imaging of biological samples using open-source DLHM setups based on 3D-printable architectures. However, these proposals inherit the disadvantages of conventional 3D printing, such as the limited resistance to mechanical stress, structural degradation due to humidity, and limited dimensional accuracy of parts. These properties severely limit their potential applicability in field analyses and PoCT. In contrast, this device proposes an aluminum-based structure that provides robustness, mechanical stability, and structural strength to the DLHM, at the expense of partially increasing the fabrication cost. The aforementioned devices also differ in their available degrees of freedom: the former has adjustable distances in both the transversal and axial directions, while the latter uses fixed axial distances and does not include any transversal-movement consideration. The herein proposed device also considers fixed axial distances, which severely alleviate alignment needs and give higher stability to the system, but it also includes a transversal displacement system that allows the fine positioning of the object at the sample plane. It is worth noting that, unlike the displacement systems reported by Tobón-Maya, the metal-based components have a smoother operation than the high-rugosity 3D-printed displacers. Additionally, to alleviate the restrictions imposed by fixed axial distances, the proposed design also presents the notable feature of a modular structure, like the design by Amann. This property means that the device can be easily adapted to adjust or replace the illumination source, the sample displacement system, or the camera, as required. Finally, regarding the illumination sources, the design by Amann uses either a fiber-coupled Blu-ray laser diode or a pinhole-filtered light-emitting diode (LED) while the one by Tobón-Maya implements an aspheric-lens-coupled laser diode. In this device, the point source is produced with a laser diode coupled to an optical fiber with a cone-shaped end. As will be presented in the later sections, using this illumination allows the selection of the desired numerical aperture by changing the processing time of a chemical etching process [17] without further modifying the bill-of-materials, thus giving the microscope enhanced adaptability beyond the installation flexibility, enhanced durability, and low-weight implementation attributable to fiber-based sources.

## Hardware description

**Fig. 1:**
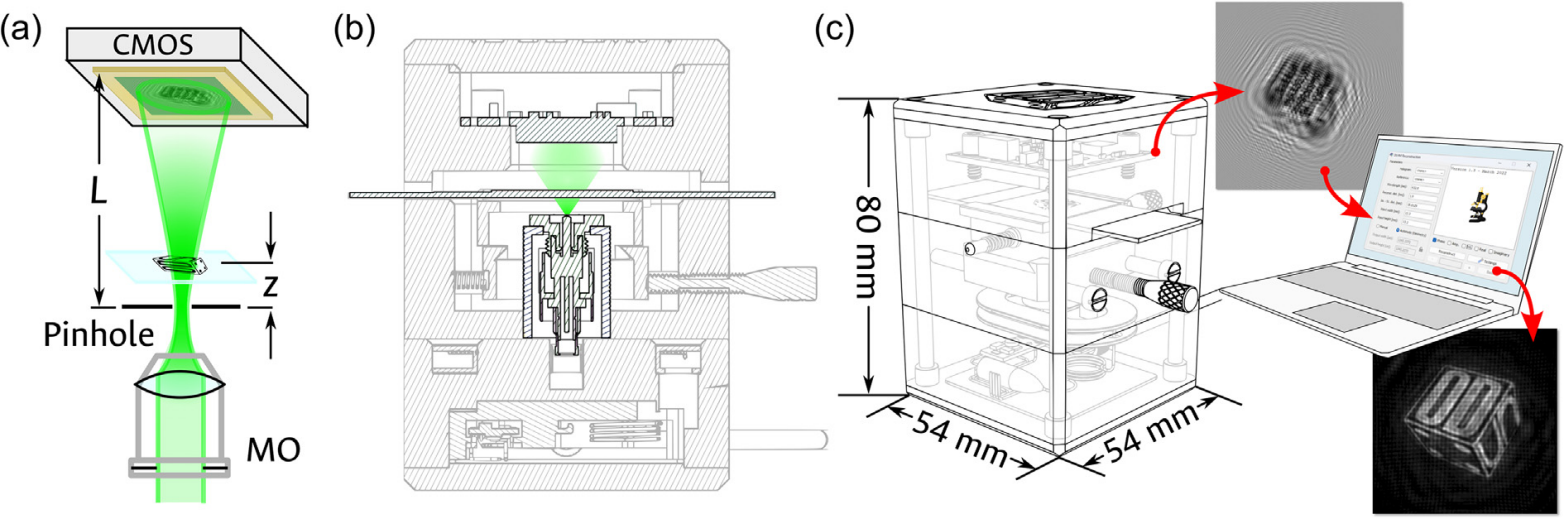
Digital Lensless Holographic Microscope (DLHM) optimized for the visualization of blood smear samples. (a) General DLHM recording configuration. (b) DLHM recording setup in the proposed device. (c) Workflow for recording and reconstruction of the DLHM device.

The traditional DLHM recording design is shown in panel (a) of Fig. 1. The spherical wavefront is generated by focusing laser light into a pinhole with aperture dimensions close to the illumination wavelength. It is then used to illuminate the microscopic sample placed at a distance z, which projects its diffraction pattern into a digital sensor located at a distance L, with all the distances measured from the point source. However, given the needed applications in PoCT, pinhole-based point sources are severely limiting as they require careful alignment and mechanical stability. Thus, the selected source is an optical fiber whose end has been modified with a cone-shaped tip. As demonstrated in Ref. [17], the modified fiber tip acts as a coherent point source, which generates the required spherically-divergent illumination for the free space magnification of the sample’s diffraction pattern. The use of this source, instead of the traditional pinhole architecture, ensures a higher sturdiness of the system and eliminates the need for careful and challenging alignment of the components. Additionally, the implementation of a cone-shaped optical fiber as the illumination source allows the resolution to be tailored and endows the DLHM with some advantageous features over the pinhole illumination system, such as installation flexibility, enhanced durability, low weight, and ease of light coupling.

The modified setup is thus shown in panel (b) of Fig. 1, in a section view of the proposed device. As will be shown in the following sections, while this system can move the sample transversally with the side knobs, the axial distances (i.e., ‘z’ and ‘L’) are fixed to further waive the need for alignment. Consequently, while the distances can be tuned in the available design files before fabrication, a given manufactured device is functionalized for a specific set of sample properties. For the case presented in this contribution, all the operational parameters and fixed distances have been selected for the analysis of blood samples. The object of main interest in a human blood-smear analysis is commonly the erythrocyte, or red blood cell (RBC). As these cells generally have sizes that range between 6 and 8 µm in a healthy subject, the proposed device must be able to adequately record, retrieve, and resolve objects within this size range. To ensure such imaging capability, the design was required to achieve a transversal resolution of 1 µm, a magnification of the diffraction pattern that allows the representation of an RBC in at least 50 pixels at the object plane, and full illumination of the digital sensor. The first factor, which guarantees enough resolution to retrieve the overall morphology of the RBCs, is determined in DLHM by the well-known relation [13] d=λ/(2NA), where d is the minimum resolvable distance, λ is the illumination wavelength and NA is the numerical aperture of the system. The second factor, which allows an adequate digital representation of the cells in the retrieved image, is given by the relation between the camera and sample distance to the point source as [13], [18] M=L/z, with M being the geometrical magnification of the diffraction pattern into the sensor. Similarly, the third factor guarantees that the numerical aperture of the illumination at least exceeds the effective aperture of the recording setup such that the full capacity of the detector is utilized; by the geometry of the setup, this condition is fulfilled if $\mathrm{NA}\geq \sin\left(\mathrm{atan}\left(W/2L\right)\right)$, with W being the width of the recording sensor.

To apply these factors effectively, the recording sensor must also be considered. The most common low-cost board-level cameras, such as the ones extracted from CCTV cameras, massive-production CMOS sensors, and even open-source-targeted cameras like the Raspberry Pi Camera module, implement formats between 1/2.3″ and 1/3.2″ with pixel sizes between 1.4 and 2.2 µm. Thus, an average sensor size of 6 mm side length with 2 µm pixels was considered for the design. For such a pixel size, the representation of an 8 µm RBC in at least 50 pixels would require a magnification factor of 12x. If the z distance is then taken in its lowest-limit value of 1 mm, corresponding to the average thickness of a standard microscope slide, the second factor sets L at approximately 12 mm. For this illumination distance, guaranteeing the full coverage of the sensor’s 6 mm length would require a NA of, at least, 0.25. Consequently, any value between 0.3 and 0.5 would be acceptable, and values between 0.4 and 0.5 are desirable. For the critical-limit value of 0.25, the fulfillment of the first factor is also verified, yielding an expected resolution of approximately 1 µm under an illumination wavelength of 533 nm. While the use of shorter wavelengths would improve the achievable resolution, it also increases the possibility of affecting the cells; thus, the use of green light serves as a compromise between resolution and minimal phototoxicity. In summary, the device is functionalized to work with an illumination distance (L) of approximately 12 mm, a sample distance (z) of approximately 1 mm, and requires an illumination source with a numerical aperture between 0.4 and 0.5. However, following the aforementioned reasoning, these parameters could be adapted as needed for other application cases.

Panel (c) of Fig. 1 shows a summary of the workflow: the compact device, with a 54 mm × 54 mm × 80 mm (W × D × H) volume, produces a DLHM hologram that is digitally recorded in any accompanying computer. All the digital communication and energy supply between the computer and the device is handled through a single USB connection, allowing a simplified operation. Once available on the computer, the hologram can be digitally reconstructed with the open-source and user-friendly DLHM plugin for ImageJ [19], which can be freely accessed at Ref.[20].

In summary, the proposed device:

• Uses a simple and compact lensless microscopy principle, allowing its cost-effective and robust implementation.
• Employs a single USB connection for both digital communication and energy supply, simplifying its use.
• Replaces the traditional pinhole-based DLHM point source with a conical-tipped fiber, waiving the daunting alignment and mechanical stability requirements.
• Implements fixed axial distances and adjustable transversal position, thus functionalizing its operation for the desired sample case while still allowing scanning of the field of view.
• Follows a modular design, easing its assembly and, if desired, customization.

## Design files summary

The components listed in Table 1 consider both metal components for CNC fabrication and polymer pieces for FDM 3D printing. However, if the overall resistance of the device is not application-critical, most of the metal pieces can also be 3D printed in polymer materials as their functionality is mostly structural.

**Table 1:** Design files summary.

Design file name	File type	Open source license	Location of the file
Camera module block	CAD file	CC BY-SA 4.0	https://osf.io/y9m7e
Sample module block	CAD file	CC BY-SA 4.0	https://osf.io/yk86a
Illumination module block	CAD file	CC BY-SA 4.0	https://osf.io/fzbg3
Sample X-Carrier	CAD file	CC BY-SA 4.0	https://osf.io/axvdc
Sample Y-Carrier	CAD file	CC BY-SA 4.0	https://osf.io/km73a
Carrier guide rod	CAD file	CC BY-SA 4.0	https://osf.io/u3sqx
Restitution pin	CAD file	CC BY-SA 4.0	https://osf.io/4vj8k
Positioning screw	CAD file	CC BY-SA 4.0	https://osf.io/gs27c
Laser Holder Cage	CAD file	CC BY-SA 4.0	https://osf.io/nuvyc
Fiber coiling support	CAD file	CC BY-SA 4.0	https://osf.io/3z6c2
Fiber port support	CAD file	CC BY-SA 4.0	https://osf.io/9skfw
Bottom cover	CAD file	CC BY-SA 4.0	https://osf.io/ekq8w
Assembly guide rod	CAD file	CC BY-SA 4.0	https://osf.io/px764
Top cover	CAD file	CC BY-SA 4.0	https://osf.io/wx3pc
Assembly model	Reference CAD	CC BY-SA 4.0	https://osf.io/6gzev
Assembly of fiber holder	Reference CAD	CC BY-SA 4.0	https://osf.io/du7fm

## Bill of materials summary

All the required components for the fabrication of the device are summarized in {Table 2}.

**Table 2:** Bill of materials.

Designator	Component	#	Cost per unit [USD]	Total cost [USD]	Source of materials	Material type
**Camera module**						
C1 – Camera block	Camera module block	1	12	12	https://www.metalsdepot.com/aluminum-products/aluminum-square-bar	Metal
C2 – CMOS	5 MP Digital Sensor	1	50	50	https://www.amazon.com/SV-usb500-W05g-Series/dp/B09TVLGNT3	Non-specific
C3 – M3 screws	M3 screw	4	0.16	0.64	https://www.mcmaster.com/93640A124/	Polymer

**Sample module**						
S1 – Sample block	Sample module block	1	12	12	https://www.metalsdepot.com/aluminum-products/aluminum-square-bar	Metal
S2 – X-Carrier	Sample X-Carrier	1	6	6		Metal
S3 – Y-Carrier	Sample Y-Carrier	1	6	6		Metal
S4 – Carrier rod	Carrier guide rod	2	2.5	5	https://www.metalsdepot.com/aluminum-products/aluminum-round-bar	Metal
S5 – Restitution pin	Restitution pin	1	2.1	2.1		Metal
S6 – Restitution spring	Restitution spring	2	3	6	Generic	Metal
S7 – Positioning screw	Positioning screw	2	2.6	5.2	https://www.metalsdepot.com/brass-products/brass-round-bar	Metal
S8 – Fiber support	Fiber port support	1	3.3	3.3	https://www.metalsdepot.com/aluminum-products/aluminum-round-bar	Metal
S9 – Fiber connector	Fiber FC/PC connector	1	1.25	1.25	https://www.fiberinstrumentsales.com/fis-fc-mm-adapter-bronze-sleeve-square-flange-2-piece-body.html	Metal

**Illumination module**						
i1 – Illumination block	Illumination source module block	1	12	12	https://www.metalsdepot.com/aluminum-products/aluminum-square-bar	Metal
i2 – Laser cage	Laser cage	1	7.5	7.5	FDM 3D printing of PLA or ABS filament	Polymer
i3 – Coiling support	Fiber coiling support	1	9	9		Polymer
i4 – Laser diode	Fiber-coupled laser diode	1	25	25	https://www.aliexpress.com/item/32844735300.html	Other
i5 – Driver	Laser driver	1	1	1	https://aliexpress.com/item/1005002515570354.html	Other
i6 – USB Cable	USB Cable	1	5	5	Generic	Non-specific

**Assembly components**						
A1 – 1/4″ magnet	1/4″ Neodymium magnet	8	0.25	2	Generic	Metal
A2 – 1/8″ magnet	1/8″ Neodymium magnet	8	0.25	2		Metal
A3 – Top cover	Top cover	1	7	7	FDM 3D printing of PLA or ABS filament	Polymer
A4 – Bottom cover	Bottom cover	1	7	7		Polymer
A5 – Assembly rod	Assembly guide rod	4	2.3	9.2	https://www.metalsdepot.com/steel-products/steel-round-bar	Metal
A6 – Power LED	Power indication LED	1	0.2	0.2	https://www.adafruit.com/product/779	Non-specific

## Build instructions

### Gathering the required components

All the required components can be either commercially acquired or fabricated from the supplied design files. Table 3 summarizes the suggested preparation for each of the components listed in the Bill of Materials.

**Table 3:** Suggested preparation of components.

**Designator**	**Suggested preparation**	**Alternative**
**Camera module**		
C1 – Camera block	CNC machining from aluminum block.	3D printing in polymer.
C2 – CMOS	Commercially available. Please check the board footprint compatibility with the assembly holes in C1 and adjust the design file if needed.	Several low-cost CCTV cameras have compatible CMOS sensors. If the casing and lenses can be safely removed, the sensor could be adapted to the design.
C3 – M3 screws	Commercially available. Might need to change the thread diameter if the selected sensor for C2 has a different footprint.	

**Sample module**		
S1 – Sample block	CNC machining from aluminum block.	3D printing in polymer.
S2 – X-Carrier		
S3 – Y-Carrier		
S4 – Carrier rod	CNC or conventional machining from round aluminum bar.	Commercially available components in aluminum or steel can be found in similar dimensions and be adapted to the required size.
S5 – Restitution pin		
S6 – Restitution spring	Commercially available.	
S7 – Positioning screw	CNC or conventional machining from round brass bar.	Commercially available components can be found in similar dimensions.
S8 – Fiber support	CNC or conventional machining from round aluminum bar. The internal diameter must match the central diameter of S9 to allow their press-fitting.	
S9 – Fiber connector	Commercially available.	Also found in a square profile; it is recommended to partially modify its contour into a circle to avoid internal collisions.

**Illumination module**		
i1 – Illumination block	CNC machining from aluminum block.	3D printing in polymer.
i2 – Laser cage	3D printing in polymer.	
i3 – Coiling support		
i4 – Laser diode	Commercially available. Must be modified to produce the conical shape in the fiber tip, as described below.	
i5 – Driver	Commercially available.	
i6 – USB Cable		

**Assembly components**		
A1 – 1/4″ magnet	Commercially available.	Other diameters can be used if the design files (A3, A4, i1, and C1) are modified accordingly.
A2 – 1/8″ magnet		
A3 – Top cover	3D printing in polymer.	
A4 – Bottom cover		
A5 – Assembly rod	CNC or conventional machining from round steel/stainless-steel bar.	Commercially available components can be found in similar dimensions and be adapted to the required size.
A6 – Power LED	Commercially available.	

### Preparing the cone-shaped fiber tip

While the fiber-coupled laser diode (component i4) could be directly used as the illumination source, the resulting spherical wavefront would be limited by the numerical aperture (NA) of a plane fiber, which is commonly between 0.1 and 0.15. As mentioned in section 2, ensuring that the illumination fully encompasses the digital sensor under the proposed distances requires a numerical aperture of, at least, 0.25. This is also the lowest possible value to achieve a 1 µm resolution with 533 nm light. If the plane fiber were to be used, the digital sensor would be underfilled by the available illumination at the design distances, and the maximum possible resolution of the system would be limited to approximately 2 µm. Thus, ensuring the usability of the device for the observation of red blood cells requires the use of NA values between 0.3 and 0.5, with the range of 0.4 to 0.5 being desirable. To increase the NA value, and thus the resolution capability of the system [21], the fiber tip can be modified into a conical shape as demonstrated in Ref. [17]. There are multiple ways in which such alteration can be achieved [22], [23], [24]. For the reported device, a chemical etching procedure is followed under what is commonly known as Turner’s method [24]. Such an approach requires the immersion of the uncoated fiber tip into a two-phase medium of hydrofluoric acid (HF) and a protective oil layer. While it can be easily implemented in a laboratory setup, the open-source project “*Cost-effective and 3D printable dip-etching device for cone-shaped optical fiber tip fabrication*”, available at Ref. [25], has the design of a simple device that allows the implementation of such a setup with off-the-shelf low-cost labware.

***WORD OF CAUTION:****Before employing a chemical etching method, it is of utmost importance to verify and recognize the potential risks associated with manipulating HF. Solutions of HF are colorless, acidic, and highly corrosive. They represent a risk for potential poisoning and injury under inadequate manipulation procedures. When using HF, make sure to strictly follow all the procedures and guidelines for its manipulation, keeping at hand the Security Data Sheet (CAS 7664*–*39-3) and ensuring the safe use of Personal Protective Equipment.*

Commercially, fiber-coupled laser diodes are commonly found with an FC/PC connector at the output, but the chemical etching procedures require direct access to the uncoated fiber. One of the following considerations should thus be applied:

• Producing the conical tip on a separate single-mode optical fiber with an FC/PC connector and coupling both fibers together.
• Producing the conical tip on a separate unconnected single-mode optical fiber, removing the FC/PC connector of component i4, and splicing both fibers together.
• Removing the FC/PC connector of the commercial fiber and processing the stripped end to produce the conical tip directly on the i4 component.

For the presented device, the third case was applied as it allows the highest stability and compactness of the illumination system. Consequently, preparing the cone-shaped fiber tip requires the additional components listed in Table 4.

**Table 4:** Materials for conical fiber tip fabrication.

Material	Cost [USD]	Source of materials
Dip-etching device	4	https://osf.io/dcba5/
FC Single-mode connector	4.25	https://www.fiberinstrumentsales.com/fis-fc-simplex-connector-3–0mm-cable-singlemode-126um.html
Fixing epoxy	5	https://www.fiberinstrumentsales.com/fis-blue-dye-epoxy-2-grams.html

**Fig. 2:**
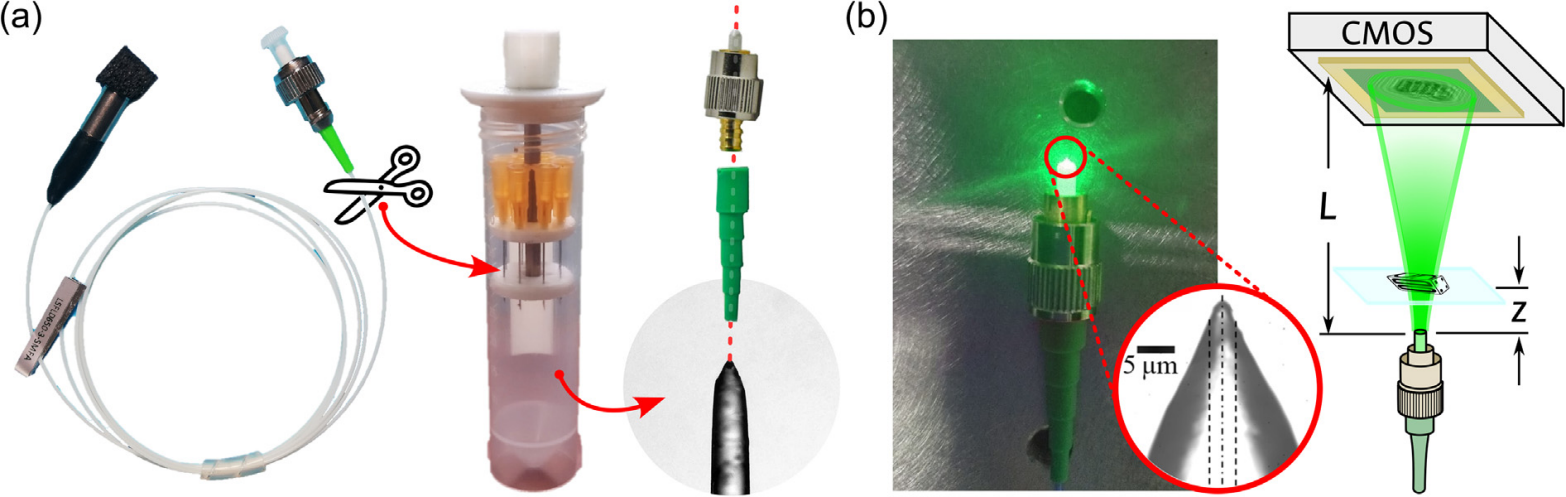
Preparation of the cone-tipped optical fiber point source. (a) Adapting a commercial fiber-coupled laser diode as a high-NA point source. (b) Modified DLHM recording setup using a cone-tipped optical fiber.

As summarized in panel (a) of Fig. 2, the fiber preparation is as follows: Initially, the connector of the fiber-coupled laser diode is removed. The protective tubing and fiber coating are then removed from the now-free end of the fiber. After cleaning and cleaving, the fiber is exposed to the chemical etching procedure via Turner’s method, either by the suggested dip-etching device [25], or any other laboratory setup. The angle to be obtained is determined by the etchant concentration and the processing time [26]. Following the procedure in Ref.[27], the desired range of NA values (namely, 0.4 to 0.5) should be obtained from HF immersions between 15 and 18 h. For this device, this cone tip was selected to have a semi-angle of 20°, which corresponds to a numerical aperture of 0.47 [26]; thus, following Ref.[27], an immersion of 16 h in a 21 % solution of HF was applied. Under the stated processing conditions, the deviation of the obtained semi-angle can be expected to be well below 10 %. If the ambient conditions are carefully controlled, this deviation has been reported to be as low as 4 %[27]. Thus, even under extreme deviation cases, the 16-hour immersion should yield results well within the desired range. Once the conical fiber tip is ready, it can be housed in a new FC/PC connector through the connectorization procedure of careful threading and fixing with epoxy (additional information on fiber connectorization can be found on the webpage of fiber suppliers [28], [29]).

After these alterations to component i4, the fiber connector can be used as a point source of high NA, allowing the simplification of the traditional DLHM setup shown in panel (a) of Fig. 1 into the scheme illustrated in panel (b) of Fig. 2. It must be noted that while most of the above-described process could be followed by a non-expert, the manipulation of HF involves health risks that should always be mitigated. The proposed method considers the use of the acid in an aqueous dilution at 21 % in very small quantities, as less than 4 mL is commonly required for the etchant preparation. However, as stated in the “word of caution”, the manipulation of this substance should always be accompanied by the strict use of all the required personal protective equipment and the immediate availability of the corresponding spill and first aid kits. Consequently, this step of the preparation should always be performed in a suitably-equipped facility by users with a general familiarity with safe laboratory practices and only after a clear knowledge of the acid’s security sheet and its associated protocols.

### Assembling the modules

After the aforementioned preparations, general construction can be pursued. The device employs a modular design, which eases its assembly and, if desired, customization. Thus, the following steps consider the independent assembly of each module and their later connection.

Camera module:

1. Take the CMOS sensor (component C2) and fix it into the Camera block (component C1) using the M3 screws (component C3) as shown in panel (a) of Fig. 3. Ensure that the cable connection port of the CMOS is placed towards the passthrough hole of the Camera block and that the sensing area is well placed in the central aperture of the block.

**Fig. 3:**
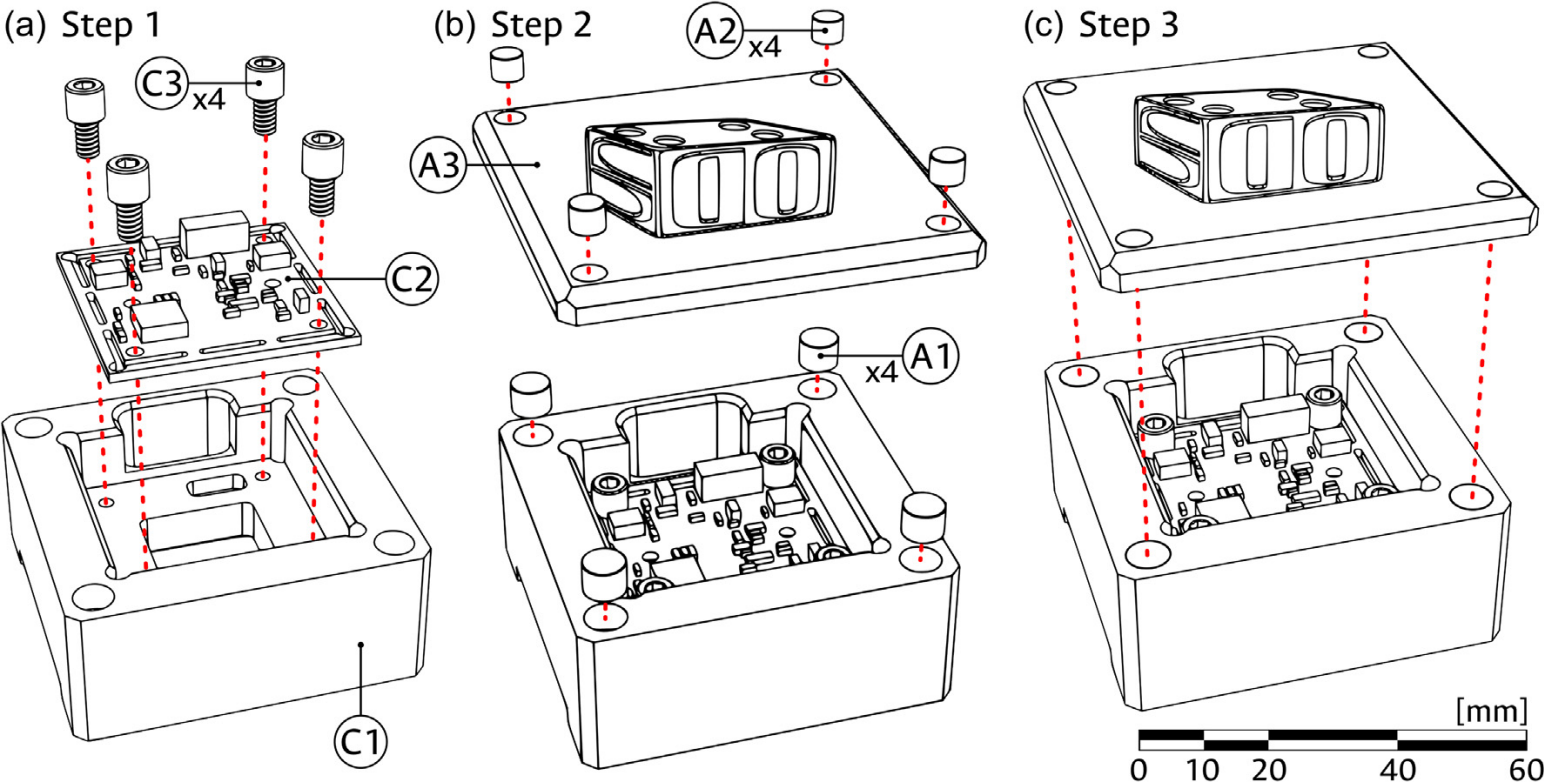
Assembly of the camera module. (a) Attaching the camera. (b) Adding magnet couplings. (c) Coupling cover to the module. The scale bar, in millimeters, applies to all panels.
2. As shown in panel (b) of Fig. 3, glue four of the 1/4″ magnets (component A1) into the corner holes of the Camera block (C1) and four of the 1/8″ magnets (component A2) into the corner holes of the Top cover (component A3). Ensure that the direction of the magnets allows the appropriate magnetic connection between the top cover and the Camera block.
3. Use the magnetic attraction between the pieces to couple the Top cover and the now assembled Camera module as shown in panel (c) of Fig. 3.

Sample module:

1. As seen in panel (a) of Fig. 4, take the Fiber connector (component S9) and press-fit it into the Fiber support (S8).

**Fig. 4:**
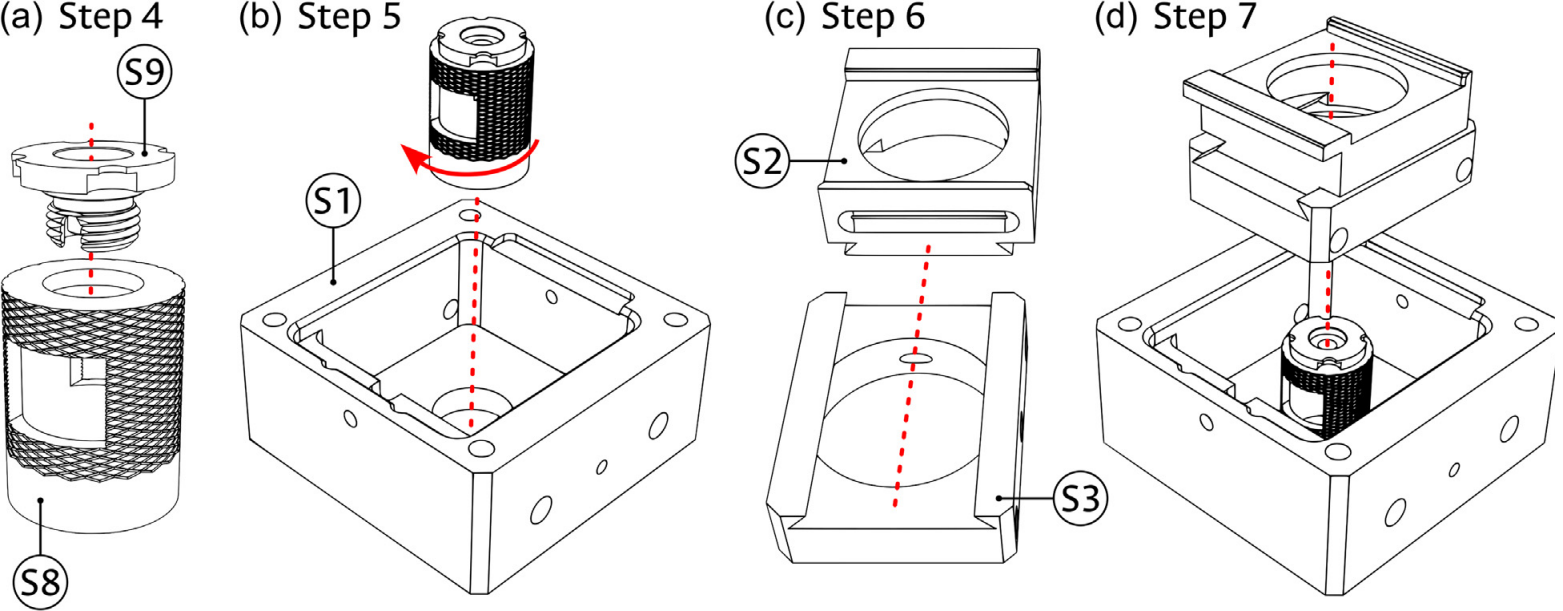
Base assembly of sample module. (a) Press-fitting of fiber port. (b) Connecting fiber port to the sample block. (c) Coupling of the sample carriers. (d) Insertion of carriers into the module.
2. Use the threaded end of the Fiber support (S8) to screw it into the Sample block (component S1), as shown in panel (b) of Fig. 4.
3. Connect the X-Carrier (component S2) and Y-Carrier (component S3) by sliding the matching dovetail features as seen in panel (c) of Fig. 4.
4. Place the connected carriers into the partially assembled Sample block in the direction shown in panel (d) of Fig. 4. Verify that the passing holes in the Y-Carrier (S3) are facing the guide rod holes in the Sample block (S1) and that the slotted feature in the X-Carrier (S2) faces the threaded feature for the insertion of the Positioning screw (component S7).
5. Introduce both Carrier rods (component S4) into the Sample block (S1) passing through the Y-Carrier (S3) as shown in panel (a) of Fig. 5.

**Fig. 5:**
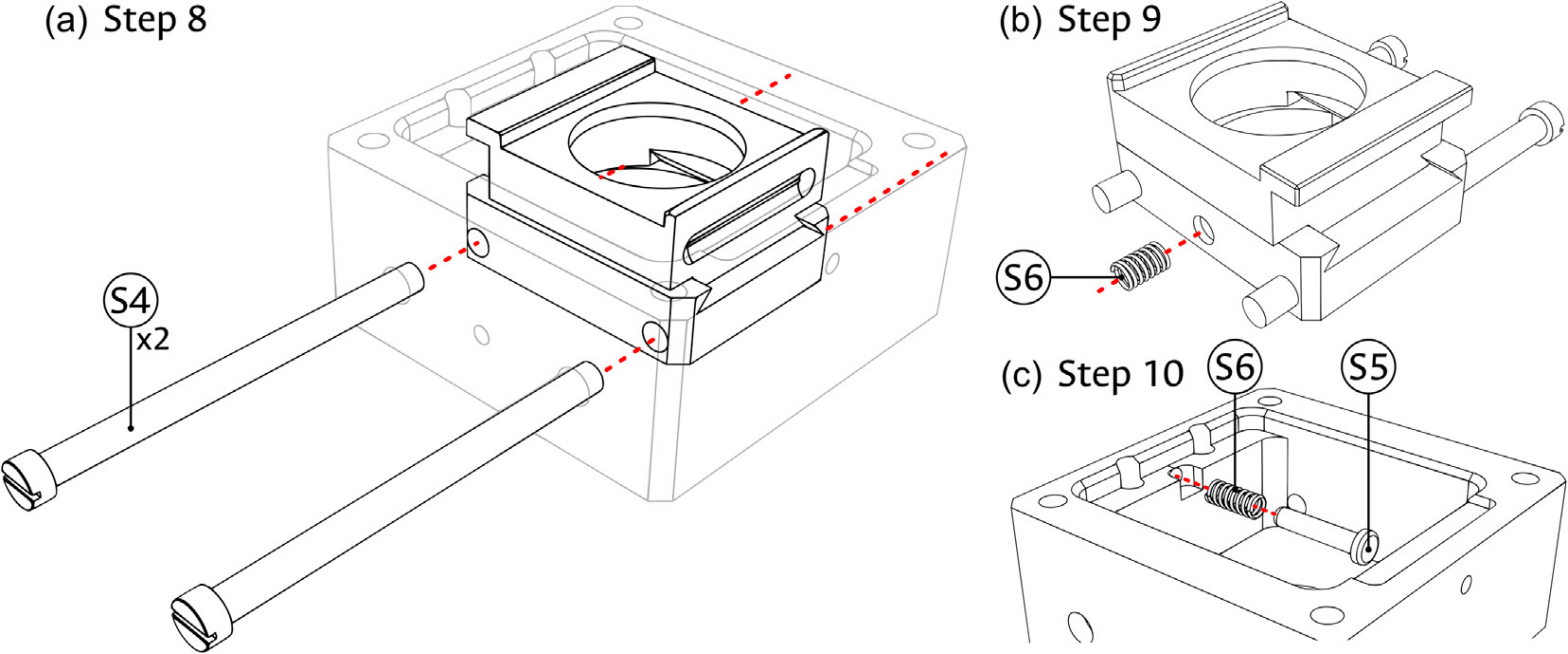
Assembly of carrier guides in the sample module. (a) Insertion of carrier guide rods. (b) Positioning of restitution spring for the X axis. (c) Positioning of restitution pin and spring for the Y axis.
6. Place one of the Restitution springs (component S6) between the Y-Carrier (S3) and the Sample block (S1), using the dimple cavity in the carrier, shown in panel (b) of Fig. 5, as placement reference and support.
7. Put the Restitution pin (component S5) through the other Restitution spring (S6), and into the Sample block (S1) using the hole in the face opposite to the threaded feature for the insertion of the Positioning screw (component S7). While the illustration of this step in panel (c) of Fig. 5 does not show the carriers, it might be necessary to partially move the X-Carrier (S2); if so, ensure that it remains inside the dovetail feature of the Y-Carrier (S3).
8. As shown in Fig. 6, introduce the Positioning screws (component S7) into the assembled Sample block (S1), using the corresponding threaded feature. The screw for the X direction should contact the X-Carrier (S3) in the center of a flat face, while the one for the Y direction should fall into the dimple cavity of the Y-Carrier (S2).

**Fig. 6:**
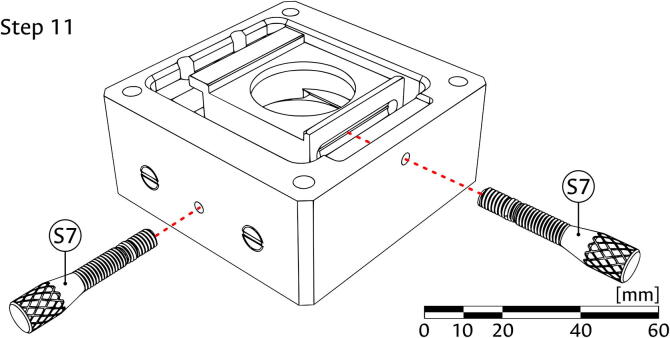
Assembly of positioning screws in the sample module.

Illumination module:

1. Place the Driver (component i5) and the Power LED (component A6) into the Laser cage (component i2) in the positions illustrated in panel (a) of Fig. 7. The driver can be fixed in place by the protruding dimples of the 3D-printed enclosure, while the LED is soldered later to the driver terminals.

**Fig. 7:**
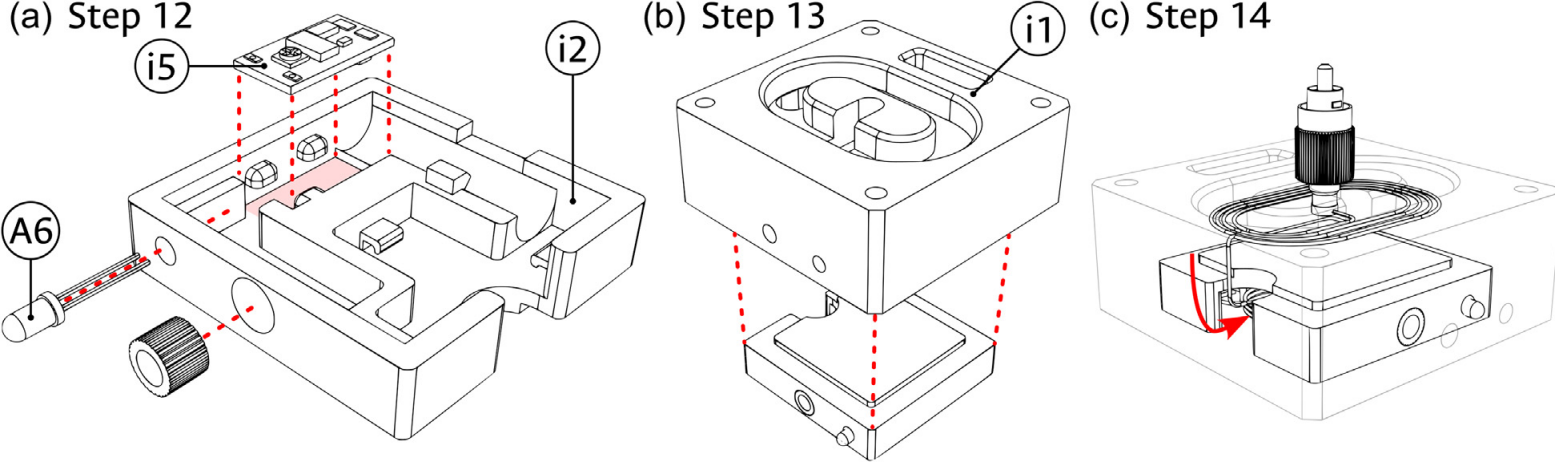
Assembly of laser holder in the illumination module. (a) Positioning of status LED, voltage driver, and cable holder. (b) Introduction of laser holder inside the module. (c) Threading the optical fiber into the module.
2. Place the partially assembled Laser cage (i2) into the Illumination block (component i1) as illustrated in panel (b) of Fig. 7. Ensure that the LED and USB apertures of the cage are facing the corresponding holes in the block.
3. Introduce the diode portion of the Laser diode (component i4) through the lateral hole in the coiling feature of the Illumination block (i1) and into the Laser cage (i2), threading the fiber as illustrated in panel (c) of Fig. 7.
4. Fix the laser diode in the laser cage as seen in panel (a) of Fig. 8. Use the protruding dimples to adjust the fiber coming through the aperture from the outside of the cage as needed.

**Fig. 8:**
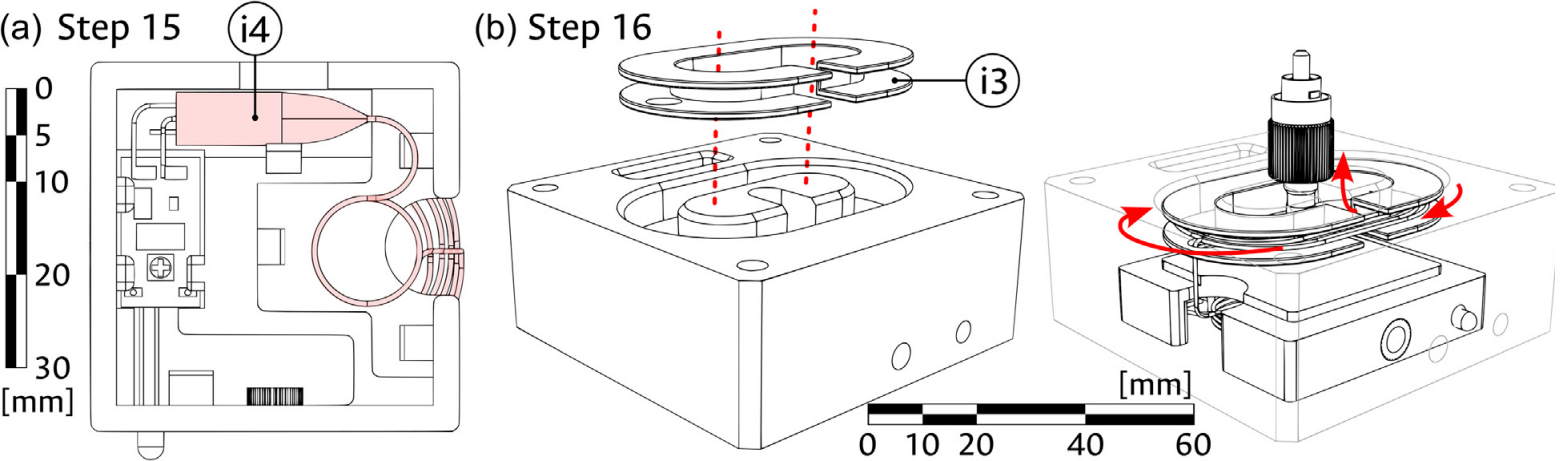
Assembly of optical fiber coil in the illumination module. (a) Adding the fiber-coupled laser diode. (b) Placing of the coiling support and the optical fiber.
5. Introduce the Coiling support (component i3) into the Illumination block (i1), using it to safely wrap the optical fiber (i4) around the coiling feature of the block as shown in panel (b) of Fig. 8. Ensure that the FC/PC connector can be placed in the central slot of the block.
6. Introduce the USB cable (component i6) through the assembled Illumination block (i1) and into the laser cage, as shown in panel (a) of Fig. 9. The four cable lines will be connected later to the corresponding terminals.

**Fig. 9:**
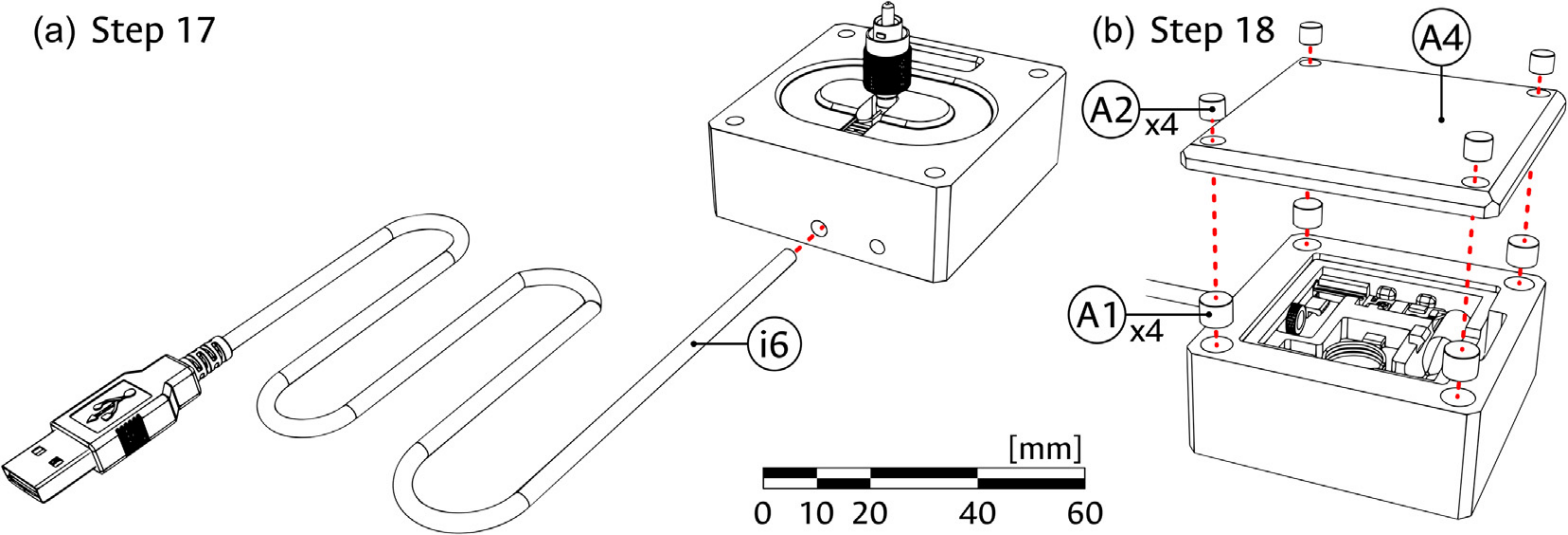
Final assembly of the illumination module. (a) Introduction of the USB cable. (b) Placing of magnets and coupling of cover to the module. The scale bar applies, approximately, to both panels.
7. As shown in panel (b) of Fig. 9, glue the four remaining 1/4″ magnets (A1) into the corner holes of the Illumination block (i1) and the four remaining 1/8″ magnets (A2) into the corner holes of the Bottom cover (component A4). Ensure that the direction of the magnets allows the appropriate magnetic connection between the cover and the block. Use the magnetic attraction between the pieces to couple the Bottom cover and the now assembled Illumination module.

### Final assembly

**Fig. 10:**
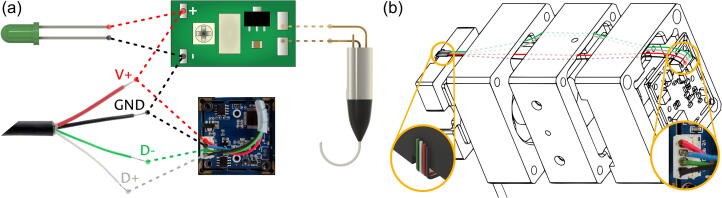
Electronic connections from the USB cable to the device. (a) Electronic connections between components. (b) Wire path between illumination and camera modules.

With each of the modules individually prepared, a full assembly can be pursued. This stage requires setting up all the electrical connections and the union of the modular blocks. When assembling the device, both operations must be done simultaneously. The electrical connections of the device are summarized in panel (a) of Fig. 10. As both the digital communications and energy supply are handled through the single USB connection, its four wires are the only external input of the system. The voltage lines, commonly found in red for the + 5 V and black for the ground, must be connected to the corresponding input terminals of the Laser driver (component i5); from these terminals, the Power LED (component A6) can also be connected, such that it turns on whenever the USB cable is supplying power to the device. The laser diode (component i4) must, in turn, be soldered to the output pads of the driver (i5), as seen in the same panel.

The data lines of the USB connection, commonly found in white for the positive signal and green for the negative one, are only needed for communication with the CMOS sensor (component C2). However, for this connection, the four USB lines must be brought from the Illumination module to the Camera module. To do so, a series of pass-through apertures are available at each block (i1, S1, and C1), allowing to pass the lines through the paths illustrated in panel (b) of Fig. 10. It is suggested to make such passings as each module is added to the assembly for easing the connections.

**Fig. 11:**
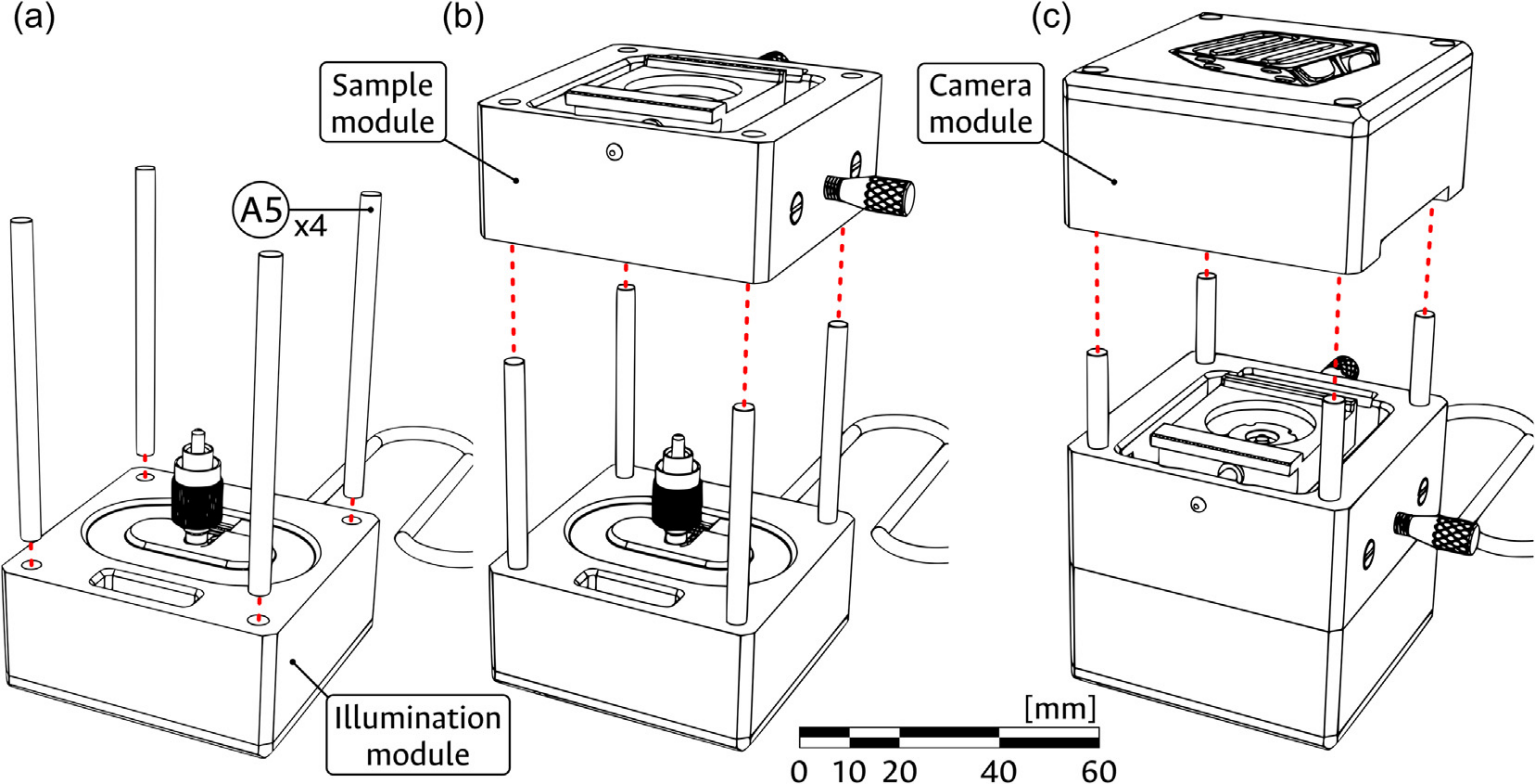
Modular assembly of the device. (a) Placing of the assembly rods. (b) Adding the illumination module. (c) Adding the camera module. The scale bar applies to all panels.

To join the modules, start by introducing the four Assembly rods (component A5) into the Illumination module. The rods are magnetically attached to the 1/4″ magnets (component A1) placed at the bottom side of the block and act as a guide and support for the attachment of the other modules. Next, add the Sample module, ensuring that the pass-through holes of both blocks are aligned, such that the USB lines can pass unobstructed. Before setting the Sample module, introduce the FC/PC connector of the Laser diode (component i4) into the Fiber support (component S8) and screw it inside the Fiber connector (component S9). Alternatively, if this proves to be difficult, the support can be unscrewed from the Sample module for the attachment of the laser diode and re-connected in place. Finally, add the Camera module, once again validating that the pass-through holes are aligned. The 1/4″ magnets (component A1) at the top of the block should magnetically attach to the rods, thus completing the assembly. The top cover can be easily removed at any time to complete the connection of the camera to the USB lines. This modular assembly procedure is illustrated in Fig. 11.

## Operation instructions

After assembly, the device can be straightforwardly operated. Initially, it must be connected to the USB port of a computer, such that digital communication can be established to the camera and the energy supply to initiate the laser can be drawn. At any point, the correct energy connection of the device to the computer can be verified by the activation of the Power LED on the Illumination module. Once connected, the camera can be accessed from the computer from any compatible webcam application. While most manufacturers may suggest a specific software, any open-source alternative can also be used. However, according to the selected CMOS sensor, the computer may need additional or updated drivers; for such cases, it is suggested to check their availability with the manufacturer or supplier of the CMOS camera. Additionally, it is highly recommended to check whether the application allows modifying the exposure time of the capture; this property allows compensation for the laser’s intensity. If such a configuration is not available, the physical power delivery can be tuned with the potentiometer in the Laser driver board. Having access to the camera in the computer, and after verifying that the laser is illuminating the sensor, the device will be ready to record holograms from blood smear samples.

**Fig. 12:**
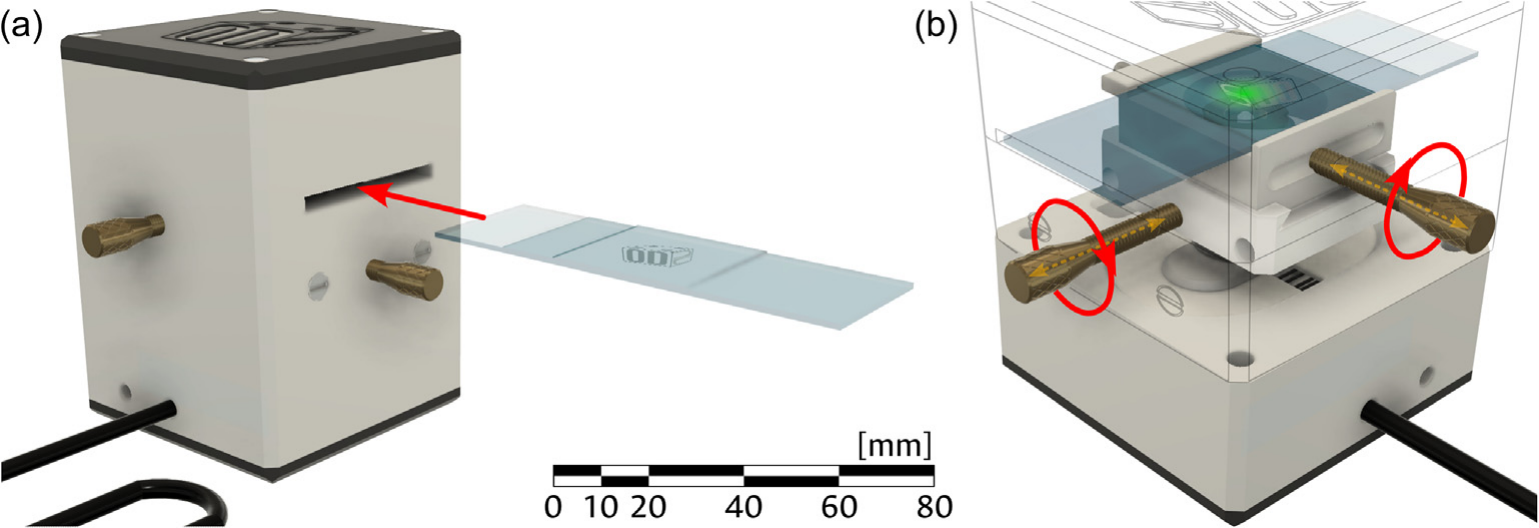
Use of the device. (a) Introduction of the sample. (b) Positioning screws move the carriers, changing the portion of the sample under inspection. The scale bar applies to both panels.

***WORD OF CAUTION:*** Avoid opening the device while the laser is powered on to prevent accidental eye exposure to the laser beam. The sample to be inspected must be placed, as in any other microscopy setup, on a glass slide. The slide is then introduced into the microscope via the lateral slots, as shown in panel (a) of Fig. 12. The placement of the specimen can be roughly adjusted by hand, but once inside the field of view can be scanned transversally by using the positioning screws, placed at each side of the device. As seen in panel (b), a rotation of either screw will cause a fine movement of the internal carriers, thus shifting the position of the sample. The webcam application can be used to follow the illuminated area in real-time, such that the inspection area can be selected. When the desired area is being recorded, the webcam application can be used to record an image of the diffraction pattern. This image constitutes the digital hologram.

Finally, the image can be brought to the open-source image processing software of ImageJ [30] which, when equipped with the appropriate plugin [19], freely available at Ref.[20], can be used to recover the complex-valued field of the sample and either its intensity or phase distributions.

## Validation and characterization

**Fig. 13:**
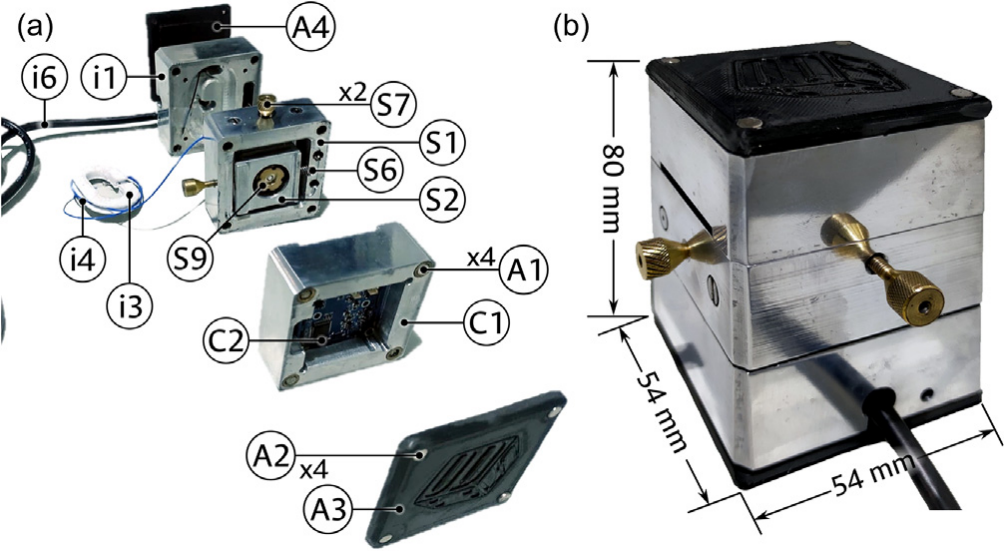
Experimental assembly of the device. (a) Exploded view of the main modular components. (b) Fully assembled device.

The proposed device was constructed following the steps and designs herein presented. To achieve the imaging of a peripheral blood smear sample, the system was equipped with a low-cost laser diode operating at 533 nm connectorized to a single-mode patch cord cable whose output connector was modified to have a cone-shaped tip of the fiber protruding from the ceramic ferrule. For the observation of RBCs, this cone tip was selected to have a semi-angle of 20°, which corresponds to a numerical aperture of 0.47 [26]. After its fabrication, the achieved numerical aperture was measured using the method described in Ref.[31], yielding 0.45; this value, while slightly lower than expected, is well within the desirable range discussed in Section 2. The point source was fixed at a distance of approximately 12 mm (parameter ‘L’ from panel (b) of Fig. 2) from a CMOS sensor with 3264 × 2448 square pixels of 1.4 µm, extracted from a commercial surveillance color camera. This sensor records the magnified diffraction pattern of the sample placed at the fixed distance of 1.2 mm (parameter ‘z’ from panel (b) of Fig. 2) from the fiber tip, which is lastly transferred to a consumer-grade laptop via USB for its processing and reconstruction with the open-source DLHM tools for ImageJ presented in Ref. [19]. Fig. 13 shows a picture of this resulting prototype, with panel (a) showing an exploded view of the modular components described in the previous sections, and panel (b) illustrating the fully assembled device.

**Fig. 14:**
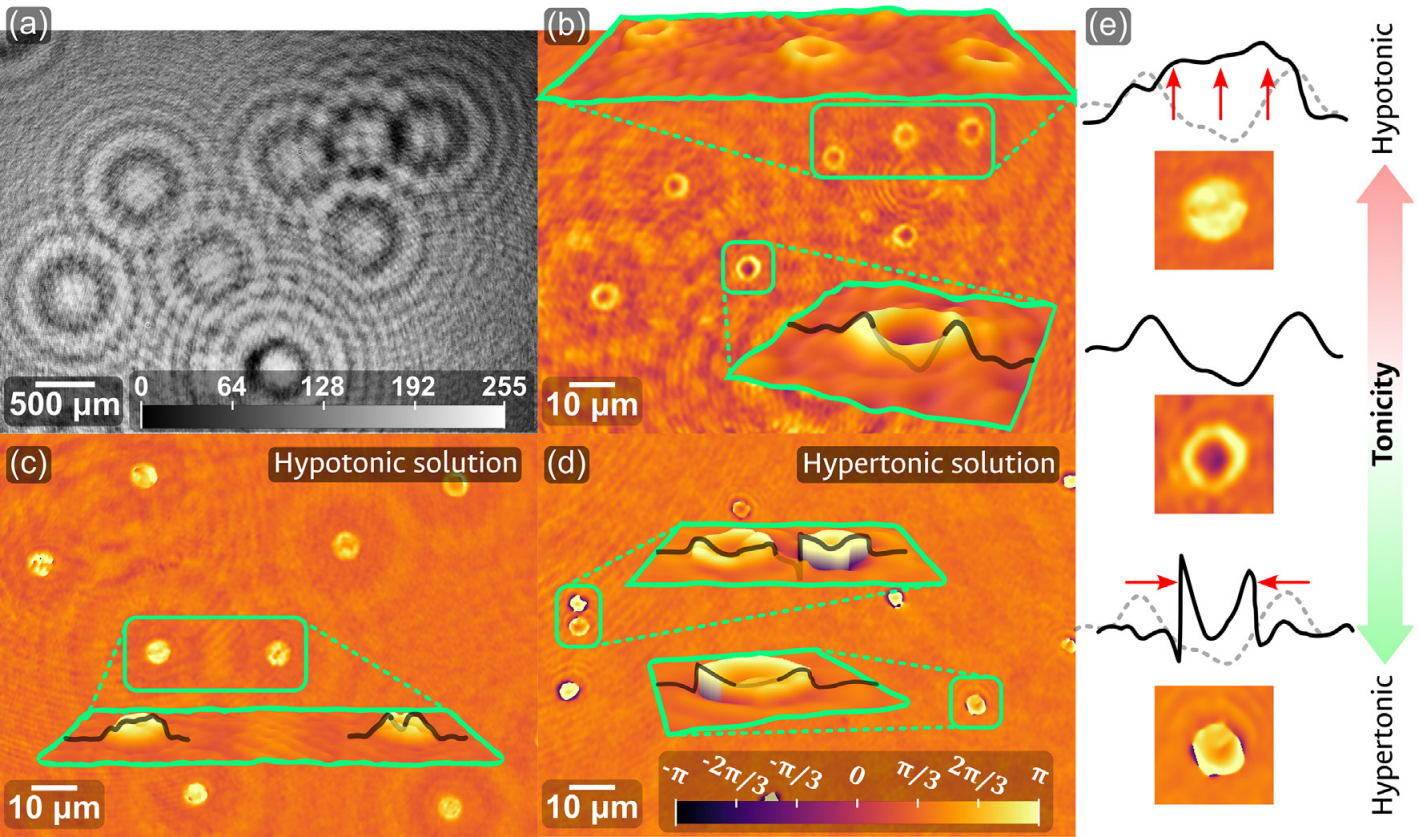
Experimental observation of blood-smear samples using the reported device. (a) 8-bit grayscale DLHM hologram of a healthy sample. (b) Phase map reconstruction of the hologram in panel a. (c) Phase map of blood in a hypotonic solution. (d) Phase map of blood in a hypertonic solution. (e) Comparison of plot profiles at different tonicity levels. The insets show 3D representations of the highlighted areas. The color scale bar in panel (d) applies to all the phase reconstructions.

This device was then used for the observation of healthy RBCs in a blood smear without any additional preparation; that is, neither dilutions nor any staining was used. The results are summarized in Fig. 14. Panel (a) shows the grayscale representation of the hologram recorded by the digital sensor for a peripheral region of the sample. As the CMOS, element C2, is a color sensor, the recorded holograms will have the color of the illumination source (here green, for the proposed 533 nm laser diode). However, the numerical processing stage ignores the color information and uses instead a grayscale version of the recording. Thus, while the image in panel (a) corresponds to the effective hologram automatically taken by the reconstruction software, a direct capture from the device’s camera would have a green hue. In this same panel, the scale bar corresponds to the sensor dimensions and the colorbar to the discrete gray levels of the 8-bit image. The characteristic diffraction pattern of multiple RBCs is readily identified on the hologram; the recorded scale of these patterns shows a significant magnification of the information, attributable to the rapidly diverging illumination impinging on the sample. As discussed in the previous sections, if the unaltered plane fiber were to be used, the numerical aperture would be limited between 0.1 and 0.15; such apertures would not be sufficient to fully illuminate the detector, and the usable information would thus be confined to a reduced region of the sensor. As a resolution of approximately 2 µm can be expected from these apertures, the RBCs would still be detected, but with a much lower definition and a smaller projected size. Such a case can be seen, for instance, in the results of Ref. [11], when a laser-coupled plane fiber is used, albeit with different axial distances. The corresponding phase map reconstruction of this hologram is shown in panel (b) of the same figure, where the RBCs ring-like shape is seen in each case. The accompanying insets of this panel show 3D surface plots of the RBCs, further showing the recovery of their characteristic shape.

**Fig. 15:**
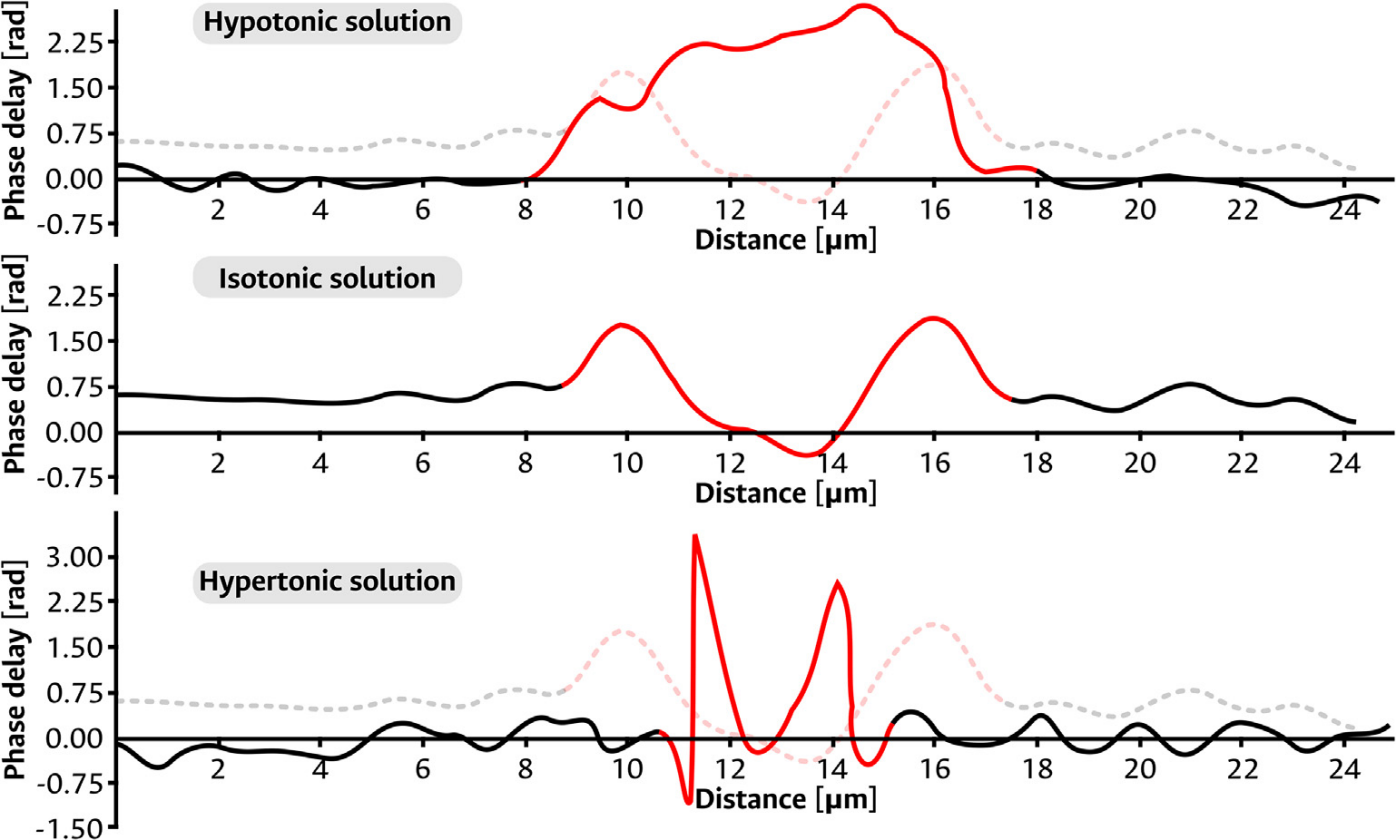
Line plots of the retrieved red blood cells’ morphology. The red portion of the plot marks the region corresponding to the RBC. In the non-isotonic cases, the background dashed line represents the healthy case, allowing a direct comparison between both cases. (For interpretation of the references to color in this figure legend, the reader is referred to the web version of this article.)

To validate the capability of the device to detect and show alterations in the morphology of the RBCs, two additional blood smears were prepared in non-isotonic media to emulate unhealthy cells. Initially, a blood sample was partially diluted in a solution of <0.6 % NaCl in distilled water. This hypotonic solution was expected to induce swelling of the cells by the osmotic flow of the surrounding water into the cell. Indeed, as can be seen from the retrieved phase information of the RBCs in panel (c) of Fig. 14, the ring-like shape of the cells is lost, being replaced by swollen cells that resemble a quasi-spherical surface. Similarly, another blood sample was then diluted in a solution of >2 % NaCl in distilled water. In this hypertonic solution, the cells were expected to shrivel and shrink due to the osmotic flow of water leaving the erythrocytes. As shown in panel (d) of the same figure, the retrieved phase map of the RBCs reveals a distorted morphology that, while partially keeping the ring-like shape, is now smaller and has sharper transitions to the background phase level rather than the smooth surfaces of healthy cells. The morphological changes just described for the hypotonic and hypertonic solutions are summarized in the profile plots panel (e) of Fig. 14, taken over the cells used for the insets of panels (b), (c), and (d). Sorted by the tonicity of the medium, these profiles show the swelling and shriveling that occurs under hypotonic and hypertonic conditions, respectively. These same alterations of the erythrocytes can be further visualized in the line plots of Fig. 15. This figure shows the well-defined profiles that can be obtained from the retrieved phase information, and how they allow the straightforward evaluation of the morphological alterations under unhealthy conditions. In each plot, the region that corresponds to the red blood cell has been marked in red. And the background dashed line that accompanies the non-isotonic cases illustrates a comparison to the healthy RBC. Once again, these plots show a clear swelling of the cells under hypotonic conditions and a shrinkage in the hypertonic solution.

The obtained results validate the capacity of this robust and compact DLHM device for the successful analysis of red blood cells in blood smear samples. The recovered phase maps show the ability of the device to retrieve morphological information of the cells, thus allowing potential imaging-based analysis of the blood health state. While its aluminum-based design involves a higher cost than a fully 3D-printed system, both in materials (metal, rather than polymer) and fabrication techniques (CNC machining, rather than fusion-deposition 3D printing), it also ensures high mechanical stability, durability, and structural strength, allowing its potential use in PoCT applications. Furthermore, the use of fine-positioning systems greatly benefits the study of microscopic specimens, as they allow a controlled scan of the sample plane. The overall dimensions of 80 mm × 54 mm × 54 mm, which constitute the device as a portable and compact system, come at the cost of employing fixed axial distances, which, while significantly reducing the alignment requirements, do restrict the adaptability of a given device to new imaging cases. Nonetheless, this limitation is partially alleviated by the modular design, which allows the straightforward adaptation of new source, sample, or camera blocks with different properties or dimensions to an existing device. Finally, the device shows the effective use of a cone-shaped fiber tip as the point source for the DLHM setup. This element of the design is recognized to be the most difficult component of the proposed device, as it requires the use of hydrofluoric acid and a suitably-equipped laboratory for its fabrication. However, it is also one of the most versatile components of the design, as its numerical aperture can be tailored as needed changing only the immersion time, and the resulting cone-shaped fiber has a high installation flexibility, enhanced durability, and minimal weight addition to the device.

## Declaration of Competing Interest

The authors declare that they have no known competing financial interests or personal relationships that could have appeared to influence the work reported in this paper.
